# The frequency modulated auditory evoked response (FMAER), a technical advance for study of childhood language disorders: cortical source localization and selected case studies

**DOI:** 10.1186/1471-2377-13-12

**Published:** 2013-01-25

**Authors:** Frank H Duffy, Yaman Z Eksioglu, Alexander Rotenberg, Joseph R Madsen, Aditi Shankardass, Heidelise Als

**Affiliations:** 1 Department of Neurology, Boston Children’s Hospital and Harvard Medical School, 300 Longwood Avenue, 02115, Boston, Massachusetts, USA; 2 Pediatric Neurology, Golisano Children’s Hospital and Upstate Medical University, 90 Presidential Plaza, 13202, Syracuse, New York, USA; 3 Department of Neurosurgery, Boston Children’s Hospital and Harvard Medical School, 300 Longwood Avenue, 02115, Boston, Massachusetts, USA; 4 Department of Psychiatry (Psychology), Boston Children’s Hospital and Harvard Medical School, 320 Longwood Avenue, 02115, Boston, Massachusetts, USA

**Keywords:** Frequency modulation, Auditory evoked response, FMAER, Cortical, Source analysis, Language disorder, Landau-Kleffner syndrome, Autism, Children, Epilepsy surgery

## Abstract

**Background:**

Language comprehension requires decoding of complex, rapidly changing speech streams. Detecting changes of frequency modulation (FM) within speech is hypothesized as essential for accurate phoneme detection, and thus, for spoken word comprehension. Despite past demonstration of FM auditory evoked response (FMAER) utility in language disorder investigations, it is seldom utilized clinically. This report's purpose is to facilitate clinical use by explaining analytic pitfalls, demonstrating sites of cortical origin, and illustrating potential utility.

**Results:**

FMAERs collected from children with language disorders, including Developmental Dysphasia, Landau-Kleffner syndrome (LKS), and autism spectrum disorder (ASD) and also normal controls - utilizing multi-channel reference-free recordings assisted by discrete source analysis - provided demonstratrions of cortical origin and examples of clinical utility. Recordings from inpatient epileptics with indwelling cortical electrodes provided direct assessment of FMAER origin. The FMAER is shown to normally arise from bilateral posterior superior temporal gyri and immediate temporal lobe surround. Childhood language disorders associated with prominent receptive deficits demonstrate absent left or bilateral FMAER temporal lobe responses. When receptive language is spared, the FMAER may remain present bilaterally. Analyses based upon mastoid or ear reference electrodes are shown to result in erroneous conclusions. Serial FMAER studies may dynamically track status of underlying language processing in LKS. FMAERs in ASD with language impairment may be normal or abnormal. Cortical FMAERs can locate language cortex when conventional cortical stimulation does not.

**Conclusion:**

The FMAER measures the processing by the superior temporal gyri and adjacent cortex of rapid frequency modulation within an auditory stream. Clinical disorders associated with receptive deficits are shown to demonstrate absent left or bilateral responses. Serial FMAERs may be useful for tracking language change in LKS. Cortical FMAERs may augment invasive cortical language testing in epilepsy surgical patients. The FMAER may be normal in ASD and other language disorders when pathology spares the superior temporal gyrus and surround but presumably involves other brain regions. Ear/mastoid reference electrodes should be avoided and multichannel, reference free recordings utilized. Source analysis may assist in better understanding of complex FMAER findings.

## Background

Spoken language is uniquely human. It facilitates complex and rapid information transfer which is essential for our species’ survival. This human adaptation allows individuals to profit not only from their own thought processes but also from the species’ pooled knowledge to which new ideas are continually contributed. Problems in individual species members’ language development, especially in childhood, constitute cause for concern and nowadays call for clinical assessment.

This paper explores the use of auditory stimulation by a frequency modulated (FM) stimulus as a potentially useful tool to assist in the assessment of developmental language disorders. The FM neurophysiological technique was described some years ago by Green et al. [1,2] with later use by Stefanatos in the demonstration of abnormality in developmental dysphasia and Landau-Kleffner syndrome (LKS) [3,4]. However, the technique failed to achieve the wide use and clinical acceptance that it may deserve. To some degree this may reflect: (1) lack of clinicians’ intuitive understanding of the FM process - most familiar to electrical and acoustic engineers and research audiologists; (2) lack of a general appreciation of the relevance of FM to language processing; (3) lack of simple, readily available equipment to perform such testing; (4) frequent observation that the maximal response amplitude to FM evoked responses is seen over the frontal-central regions, rather than over language associated temporal regions [1,2]; (5) mixed results in the literature with some authors reporting negative [5] and others positive [6,7] findings with FM stimulation in childhood language disorders, and (6) lack of data driven demonstration of FM’s utility in various language disorders.

### Unique characteristics of human speech

Human speech constitutes a unique auditory signal. For example, it is rare to confuse random background noise with human speech. A great deal is known about the specific acoustic characteristics of human language with much relevant information arising from studies of language pathophysiology. For example, Tallal demonstrated that children with developmental dysphasia manifest difficulty in their ability to accurately detect and process brief tone pairs. As intervals between tone pairs are shortened, dysphasics are significantly more adversely affected than controls. Tallal, in a series of papers, demonstrated [8-12] that language impaired children have problems processing auditory information when presented at a normal rate. This deficit reflects their inability to process the rapid acoustic changes that lie within normal speech streams. Speech contains rapid changes that must be detected in order to be comprehended appropriately [13-15].

Deficits in auditory processing speed may adversely affect the ability to perceive phonemes, brief complex sounds with rapidly changing spectral characteristics. As Stefanatos [16] summarizes: “The human auditory system is comprised of specialized mechanisms that subserve the abstraction and coding of temporal features of FM in temporally complex sounds. These mechanisms or channels are not merely concerned with the detection of a change from one frequency to another but are sensitive to the instantaneous temporal properties of frequency change such as rate, shape, direction, and periodicity of modulation [17,18]”. As an illustration of this, Stefanatos demonstrated that in an adult with pure word deafness, the evoked response to pulsed frequency modulation was substantially reduced [16].

### Frequency modulation (FM) and amplitude modulation (AM) explained

**Figure 1:**
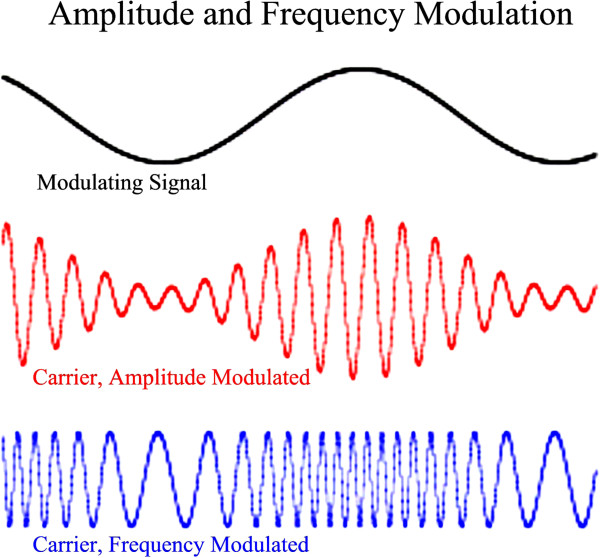
**Amplitude and Frequency Modulation Illustration.** Three sinusoidal waveforms are shown. The upper, slow, sine wave (black) is utilized to modulate or impress itself upon the lower two, faster “carrier” sine waves. When the slow modulating signal is impressed by means of alteration of carrier amplitude, the result is as shown in the middle red waveform – Amplitude Modulation or AM. If the AM modulated carrier were to be an audible signal such AM would cause it to wax and wane in amplitude while the carrier frequency would remain unchanged. In contrast if the modulating signal were to be impressed upon the carrier by means of alteration of carrier frequency, the result would be as shown in the lower blue waveform – Frequency Modulation or FM. If the carrier were to be an audible signal, such FM would cause it to rhythmically change frequency (or warble) but the carrier loudness or amplitude would remain unchanged. Human language appears to involve rapid changes in FM content of auditory signals especially important in the production and detection of phonemes.

Figure 1 illustrates the imposition of a slow signal (upper sine wave) upon a faster “carrier” sine wave by means of AM (middle red sine wave showing varying amplitude with constant frequency) and FM (lower blue sine wave showing constant amplitude with varying frequency). Although AM and FM are commonly used to modulate high frequency radio carrier signals with much lower frequency audio signals, the use of FM and AM techniques in neurophysiologic testing is specifically designed to uncover defects in cortical auditory processing which may not always be clinically evident. Details are outlined below.

### Goals

The overall goal of this paper is to demonstrate the FM’s utility in preparation for future detailed studies in various disorders of language. This paper furthermore attempts to facilitate the clinical use of the frequency modulated auditory evoked response (FMAER) by illustrating its origins within eloquent language cortex and by demonstrating temporal lobe abnormalities observed in selected childhood language disorders. The overall goal is served by five specific sub-goals: (1) To describe methods for creating stimuli useful for clinical application of the FMAER; (2) to demonstrate the scalp appearance of the FMAER in normal subjects; (3) to locate brain sites where the FMAER is generated; (4) to provide examples of the types of FMAER abnormality that have been observed in various childhood disturbances of language, specifically Developmental Dysphasia, Landau-Kleffner Syndrome, and Autism Spectrum Disorder (ASD); and (5) to increase understanding of the relevance of the presence or absence of the FMAER response in a given language pathology.

## Methods

### Study population

All data were gathered in the neurophysiological laboratories of a university affiliated (Harvard Medical School) academic medical center (Boston Children’s Hospital - BCH). The Developmental Neurophysiology Laboratory (DNL) at BCH, under the direction of the first author, maintains a data base of patients, including those referred for clinical study and those participating in research studies. The data base provides unprocessed (raw) EEG and evoked response data as well as referral and clinical information. From these studies, data were selected on a de-identified basis for three groups of subjects, neurotypical research control subjects (ages 7–20 years old), clinical subjects (ages 2–9 years old), and epilepsy surgical subjects (ages 3–20 years old).

#### Research control subjects

From the population of research studies, those containing FMAER data collected from neuro-typical control subjects were selected to determine normal FMAER findings. All control subjects selected met the following criteria: (1) Living at home and considered normal by their parents and/or living away from home to attend school and without evidence of academic difficulty; (2) without history of speech, language, or hearing difficulty; (3) without need for and use of medications at time of study; (4) with normal EEGs at time of study, i.e., containing no seizure discharges and no evidence to suggest an active seizure disorder; and (5) without history of neurological, medical, or neuropsychiatric illness or abnormality.

#### Clinical subjects

From the clinical population referred to BCH for combined EEG and evoked response (ER) evaluation, those studies were selected that included the FMAER test and a referral diagnoses specific for language disorder. This population included referral diagnoses such as learning disability, developmental dysphasia, LKS, and autism spectrum disorder (ASD). With the exception of LKS, clinical subjects with active epilepsy or frequent seizure discharges were excluded, as were patients with MRI evidence for tumors or cortical dysplasia including tuberous sclerosis. Taking medication at time of study was not an exclusionary criterion for the clinical study population. For the purpose of this report, subsets of subjects were selected whose FMAER data appear to be normal or abnormal in order to illustrate the various types of abnormality observed across the larger clinical population with language disabilities.

#### Epilepsy surgical subjects

Over the past five years, FMAER studies were requested on seven patients undergoing Phase 2 invasive evaluation for possible epilepsy surgery. Such evaluations involve initial surgical placement of subdural (cortical) grids and strips for prolonged (one week duration) electrocorticography (ECoG). Requests for cortical FMAER studies were made in order to complement direct cortical stimulation and functional magnetic resonance imaging (fMRI) in the localization of eloquent auditory cortex prior to removal of epileptogenic cortex when epileptic foci were tentatively located within the temporal lobes [19-21]. The reasons for FMAER requests included situations where language evaluation by direct cortical stimulation was considered to be potentially unreliable due to patient immaturity, behavioral difficulty, language disorder, and/or clinician-patient language barrier.

#### Institutional review board approvals

All control subject families, and subjects as age appropriate, gave informed consent in accordance with protocols approved by the Institutional Review Board (IRB) of BCH in full compliance with the Helsinki Declaration. Subjects, who had been referred clinically, were studied under an IRB protocol in full compliance with the Helsinki Declaration, which required data de-identification without the requirement for informed consent.

### The clinical stimulus

A carrier sine wave at 1000 Hz is frequency modulated by a slower 10 Hz sine wave causing the frequency of the carrier wave to shift (“deviate”) between 960 and 1060 Hz at the 10 Hz rate thereby producing a warbling tone. Next the 10 Hz sine wave is amplitude modulated by a slower 4 Hz sine wave such that the warbling (FM modulation) is sinusoidally turned on and off (AM modulated) at the 4 Hz rate. This process causes the 10 Hz “warbling” of the 1000 Hz sine wave carrier to be sinusoidally turned fully on and off (100% modulation) at 4 Hz. By setting a trigger pulse to the start of each second of 4 Hz signal, signal averaging can be performed in order to obtain an ER, namely the FMAER, that is time locked to the 4 Hz AM modulation of the 10 Hz FM modulation, i.e., to the turning on and off of the FM. Typically 300–1000 trigger pulses are averaged over an epoch of 1000 msec. In normal subjects a resulting one second FMAER manifests a 4 Hz sine wave when recorded from active scalp or cortical electrodes. This process broadly corresponds to the original description of Green et al. [2]. The stimulus’ sound pressure level is held at approximately 78db SPL, measured at the ears and is delivered by either earphones or nearby speakers depending upon the environment and subject preference/tolerance. The continuous stimulation tone produced as described above has been found to be suitable for behaviorally challenged patients and appears easier to employ clinically than standard tone pulses or clicks often used to form click evoked auditory evoked responses (AER). The more abrupt amplitude variations associated AER clicks often appear to upset certain clinical patients. Furthermore, click stimuli often produce movement artifact in lock-step with the stimulation, e.g., time-locked eye blinks and/or muscle activity bursts. Signal averaging does not diminish such time-locking as the artifact occurs in time with respect to the stimulation. Sinusoidal variation, as employed in the FMAER, in contrast, does not appear to induce significant time-locked artifact.

At time of clinical study, FMAERs are first formed from successive thirds of all stimuli which, when separately evaluated, allows assessment of response consistency. If responses are similar across all three thirds, a global average is formed for interpretation. If such consistency is not observed, more data are collected to improve the signal to noise ratio. Bandpass filtering (typically 2–12 Hz) may be helpful when the background EEG is unusually high in amplitude or shows considerable artifact.

Quantification of the 4 Hz response may, at times, be useful, for example, when following a patient with serial studies over time or when performing a group-comparison study that involves multiple subjects. Aside from basic peak-peak or root mean square voltage (Vrms) measurements of appropriately averaged and filtered ERs, a spectral analysis of the ERs may also be performed, utilizing the magnitude of the 4 Hz component as the response measure [22].

The Chirp2™ Signal Generator (Mind Spark Inc., 172 Washington St, Newton, MA 02458 USA), a small stand-alone battery operated device, was employed to perform all aspects of FMAER signal generation and trial marker formation. The slow, 4 Hz AM signal was selected based upon Green’s original work and the finding by Talcott et al. [7] that “…40% of the variability in children’s phonological and reading skills can be predicted from their sensitivity to 2 Hz frequency modulated…tones …(which does)…not hold for (faster) 240 Hz FM.”

### Recording conditions, data collection, and initial data processing

The EEG data for clinical subjects utilized in this study were gathered in the clinical laboratory (Clinical Neurophysiology Laboratory of BCH) from 30 scalp channels via gold cup electrodes applied with collodion after careful measurement by a registered EEG technologist. A 31st channel carried a trial marker indicating onset of the 4 Hz signal that modulated the 10 Hz FM. A 32nd channel carried eye movement and blink artifact information. Data were digitized at 256 Hz after amplification by a Cardionics™ 32 channel EEG amplifier (Cardionics Inc. 910 Baystar Blvd, Webster, TX 77598 USA) set to 1–100 Hz pass band. The 30 EEG scalp channels used included the following: FP1, FP2, F7, F3, FZ, F4, F8, FC5, FC1, FC2, FC6, T7, C3, CZ, C4, T8, CP5, CP1, CP2, CP6, P7, P3, PZ, P4, P8, O1, OZ, O2, TP9, TP10. Data for the FMAER were gathered over 5–20 minutes with additional time allowed for rest breaks as needed. The patient and a parent, when behaviorally indicated, were together in a sound shielded room adjacent to the recording equipment and visible to the technologist. Off-line, the EEG data and accompanying trial markers were visually evaluated and epochs containing excessive eye-blink, muscle and movement artifact were marked for removal from subsequent analysis. Signal averaging, EEG review, and topographic mapping of the FMAER were performed using Nicolet™ software (Nicolet Biomedical Inc., 5225 Verona Rd, Madison, WI 53711 USA).

The EEG data for research subjects were gathered in the research laboratory (DNL) utilizing an EGI™ 128 channel net system (Electrical Geodesics Inc, 1600 Millrace Drive, Suite 200, Eugene, OR 97403 USA) along with a single information channel dedicated to the stimulus trial marker as for the clinical laboratory. Conductive jelly was employed with the research net system instead of salt soaked sponge electrodes which more likely result in electrode artifact and/or saline (conductive) inter-electrode bridges when data collection extends over an hour. The research subject and a parent, as indicated, were together in a set-up similar to the clinical laboratory, namely a sound and electronically (Faraday) shielded chamber adjacent to the recording equipment and visible to the technologist via a one-way mirror window. Data were sampled at either 250 or 500 Hz with 0.1-100 Hz EEG band pass. Recording times were as noted above. After recording, all research subjects with electrode nets in place underwent photogrammetry, an eleven camera based EGI system, to establish the precise position of the 128 net electrodes thereby facilitating off-line mapping to standard EEG electrode positions (noted above) for comparative purposes and also for later co-registration with either subject specific or standard MRI images. As for the clinical laboratory, research laboratory data were de-artifacted, signal averaged, and re-montaged now via BESA™ software (BESA GmbH, Freihamer Str. 18, 82116 Gräfelfing, Germany).

The EEG data from epilepsy surgery patients were obtained on the inpatient BCH Epilepsy Service from 128 channel Natus™ amplifiers (Natus Medical, Inc., 1501 Industrial Rd, San Carlos, CA 94070 USA) with 1–100 Hz bandpass, sampling at 256 Hz. One channel contained a stimulus related trial marker. Data were recorded directly from brain utilizing cortical “grids and strips” - electrodes that had been surgically placed and positioned to detect and record EEG spikes and/or seizure discharges. Recordings that contained continual or frequent seizure discharges or electrodes that had not been placed near or over the temporal regions were not included in this study. Signal averaging for this sample was performed by BESA software.

### Source analysis of scalp recorded FMAER data

Discrete dipole source analysis was performed according to the BESA manual tutorial and as recently demonstrated by Hagenmuller et al. [23] in a study of source analysis of the click auditory evoked response (AER) to stimuli of varying loudness. The key to successful AER source analysis is the ‘seeding’ of starting sources in both temporal regions. Failure to do this, i.e., starting with a single initial source, invariably results in a biologically non-existent midline source solution. Dual, bilaterally symmetrical seed sources are best when the biology suggests largely symmetrical bi-hemispheric sources as is true for the FMAER as well as the click AER. The first author’s laboratory has had extensive experience in source analysis of epileptic foci from scalp EEG recordings in children having performed more than 170 such studies over the past eight years on outpatient and inpatient epileptic patients under consideration for possible epilepsy surgery.

Source analysis results in a six dimensional solution for each calculated source. Three dimensions reflect the physical location of the source within the brain (typically shown as a small circle or dot). Another three dimensions are needed to demonstrate the orientation of the source considered as an electrical dipole (shown as a line attached to the dot representing the source location). The scalp distribution of a given source is determined by both source location and source orientation. Within the current report, when summarizing a large number of subjects in a single image and where source location is the primary consideration, the source orientation is omitted.

For any given source analysis on neurophysiologic data (typically ERs or EEG containing seizure discharges) a first step is to estimate the number of underlying sources needed to explain the scalp recorded data. There could, theoretically, be as many sources as the number of recording electrodes utilized - although there are typically far fewer. BESA utilizes Principal Components Analysis (PCA) [24] to estimate the minimal number of sources needed to represent the scalp data. For typical neurophysiological PCA a matrix (table) is formed from the data to be analyzed where the rows represent electrodes and the columns represent the ER or EEG data across the selected time epoch for each electrode. PCA breaks down the scalp electrode data matrix into a small set (reduced matrix size with fewer rows) of building blocks (principal components or factors) where every electrode’s data can be represented by a linear combination of the derived factors. The number of ‘significant’ factors is typically limited to those that explain over 1% of the total variance [25,26]. It is assumed that at least as many sources should be demonstrated as there are significant factors. However, it has been pointed out that in some circumstances there may be more sources than factors. This is, for instance, the case when two nearly symmetrical generators (sources) are primarily responsible for a single PCA-derived factor waveform. That occurs, typically, for long latency auditory evoked responses (ERs) which are simultaneously generated by both temporal lobes when responding to controlled external stimuli. It is less likely the case for epileptic discharges. All sources derived by source analysis may prove to be important as clinically determined by their location and source waveforms. However, both averaged spike samples and averaged ER data contain residual background EEG noise unrelated to the primary quest for spike or ER origins. Nonetheless if a minimal number of residual noise source locations (reflecting, for example, eye movement, alpha, muscle, etc.) are not calculated then the locations of the primary sources of interest may be spatially offset to an undetermined degree by the unaccounted/unanalyzed residual noise.

FMAER data recorded from both the 32 channel clinical and 128 channel research polygraphs were transferred to formats readable by BESA 5.3 where source analysis was performed. Source analysis findings from scalp recorded data were mapped into BESA-supplied, age group specific, averaged MRI images for the reports of source localization.

## Results and discussion

### Source analysis of scalp recorded data

**Figure 2:**
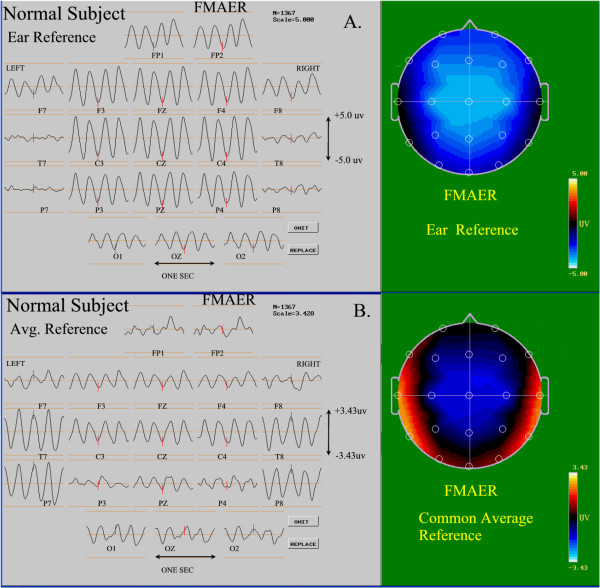
**Normal FMAER - effect of reference electrode.** The waveforms for 20 channels of a 4 Hz FMAER study are shown to the left as recorded from a neuro-typical 7 year old control subject. Electrode designations appear below each channel’s waveform which is one second in duration. An amplitude scale is shown to the right in microvolts (uV). All data are derived from the same subject. Topographic voltage maps are shown to the right at the same scale as the waveforms, with blue representing negative and red positive activity. For each map the nose is shown above, occiput below, with left ear to the left. The map is formed at the maximum negativity of electrode CZ at the waveform midpoint. The upper combined waveform and topographic map (**A**) show the display as it appears when the data are referenced to linked ears, as is commonly used. The lower display set (**B**) shows the data when the common average reference is employed, as recommended here. Note the difference in both amplitude and spatial locations of the FMAER on both the waveform and the mapping imposed by the difference in referencing.

**Figure 3:**
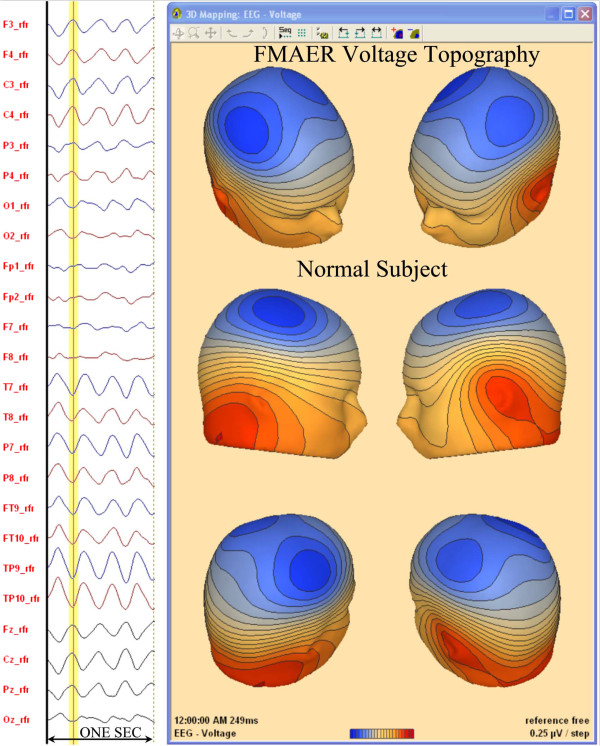
**128 Channel mapping of normal FMAER, common average reference.** The same data from the same 7 year old normal subject, shown in Figure 2, are now depicted when utilizing 128 channels (24 selected waveforms as shown to the left) with utilization of the “reference free” technique, which is quite similar to the common average reference approach (Figure 1B). The waveforms to the left are one second in length. In the 3D head maps depicted to the right, the FMAER shows a topographic distribution suggestive of two dipoles, one over each hemisphere with negative (blue) end over the central region and positive (red) end in the mid temporal region and below. The spatial topography suggests the source may be between these two scalp maxima, likely in the temporal lobes.

**Figure 4:**
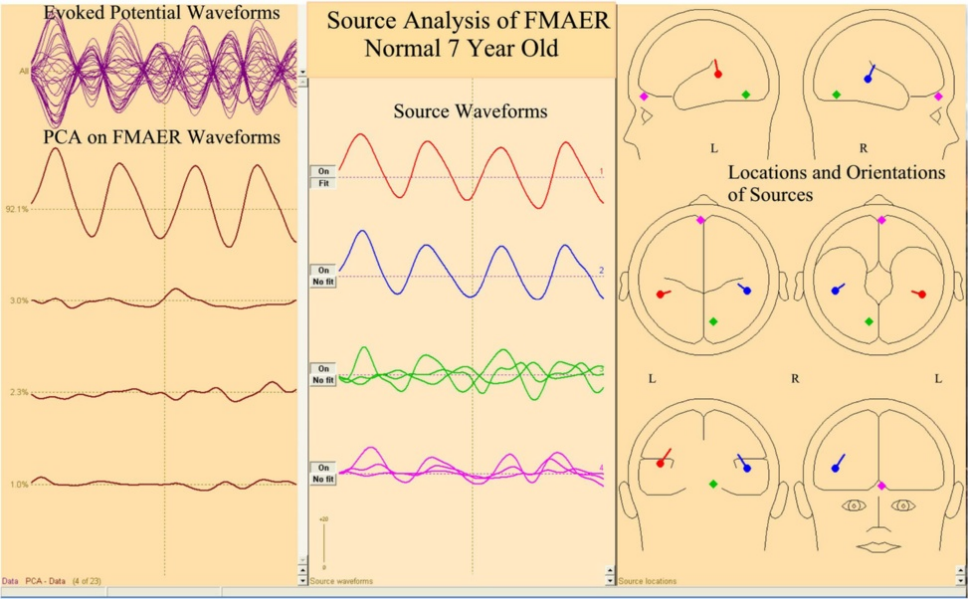
**Source analysis of a normal subject’s FMAER.** Source analysis is illustrated for the same 7 year old whose scalp data are shown in Figures 2 and 3. The time base is approximately one second for all waveforms. The top of the left pane shows all channel data overlain; below this a principal components analysis (PCA) illustrates a single, prominent, primary factor showing the expected 4 Hz sine wave. Source analysis (Hagenmuller at al. [23]) shows two major sources whose source waveforms are shown in the center pane and whose source locations and orientations are displayed in three dimensions within the right pane. Final two sources explain residual noise (Hagenmuller at al. [23]). Source waveforms and source locations are correspondingly color coded. See also Figure 5.

**Figure 5:**
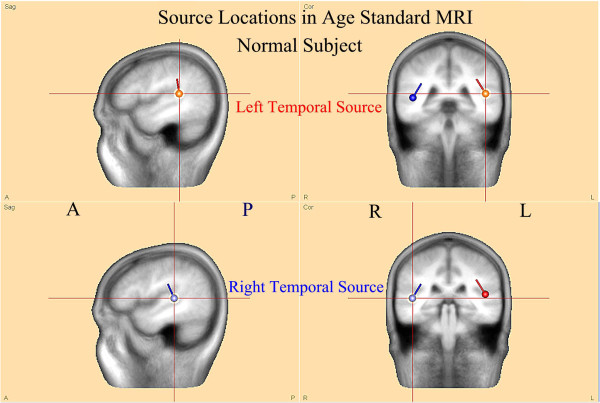
**FMAER source analysis in standard MRI atlas for age.** The sources illustrated in the right pane of Figure 4 for the 7 year old normal subject are now shown within the standard 6–8 year old BESA software supplied standard MRI. Note that both source dipoles are centered in the posterior superior temporal gyri at the lower edge of the angular gyri. Also note the source orientations, pointed towards the central vertex region superiorly and posterior inferior temporal regions inferiorly. These locations and orientations explain the scalp dipolar distributions observed in Figures 2B and 3.

Figure 2 shows a subset of twenty standard EEG channels [27] illustrating the 4 Hz FMAER recorded from a single 7 year old neuro-typical research subject with normal language function. The upper image (Figure 2A) utilizes the ears as reference point and the lower image (Figure 2B) utilizes the common average reference [28]. To the right, the corresponding topographic maps [29] are shown taken at the peak of the 4 Hz sinusoidal response waveforms. Note the effects of changing the reference technique from ears to common average. The difference between data shown from the same subject in Figure 2A and 2B reflects that ears (Figure 2A) constitute poor reference sites when the adjoining temporal lobes are active. When the ear reference is employed, the maximal FMAER response is erroneously presented in the midline central-frontal region and appears absent in both temporal lobe sites (Figure 2A). However, when the common average reference is employed, the maximal FMAER response is correctly observed in the left and right temporal regions independently (Figure 2B). Figure 3 demonstrates the FMAER topography for the same 7 year old neuro-typical research subject with normal language function now illustrated with 24 electrodes and maps, derived from 128 channel data, using reference free mapping based upon the common average approach. Figure 4 illustrates details of the source analysis of the same 7 year old neuro-typical research subject with normal language function. PCA [24,30] on the entire electrode set showed one primary, 4 Hz sine wave dominant component (top left column) which by source analysis decomposed into two major responses (top two center column) with origins in left and right posterior superior temporal gyri (right column) and with source orientation directed toward the central vertex region. The final two source components reflect primarily residual occipital and prefrontal alpha and blink activity along with small amounts of 4 Hz activity. Figure 5 shows these same data now mapped on a manufacturer (BESA) supplied age appropriate standard MRI. Note that bilateral temporal sources are in the *planum temporale* and oriented towards the vertex region.

**Figure 6:**
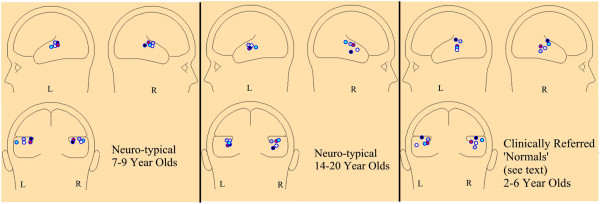
**FMAER source locations for 15 subjects.** The three panes each show three schematic head maps seen from the left, right, and posterior views. Each pane illustrates the locations of primary left and right temporal sources for the indicated groups. The colored dots remain color specific for a single subject, e.g., within each pane the red dots reflect data from the same individual subject. Each subject’s FMAER data were source analyzed, displayed in an age appropriate normalized MRI, and the results graphically transferred to the schematic head maps shown. Source location is shown without source orientation.

Figure 6 shows the results of source analyses of 15 right handed subjects including five 7–9 year old (left pane) and five 14–20 year old (center pane) neuro-typical controls. Note the tight clustering in the posterior temporal gyrus for the 7–9 year olds. The 14–20 year olds showed a similar pattern in the left temporal region with a slightly less restricted spatial distribution in the right temporal lobe. The right pane shows five 2–6 year old clinical patients with diagnoses of ADHD without clinical evidence of language dysfunction and with scalp FMAER patterns that appeared normal by visual inspection.

### Cortical FMAER from surgically placed grids and strips

**Figure 7:**
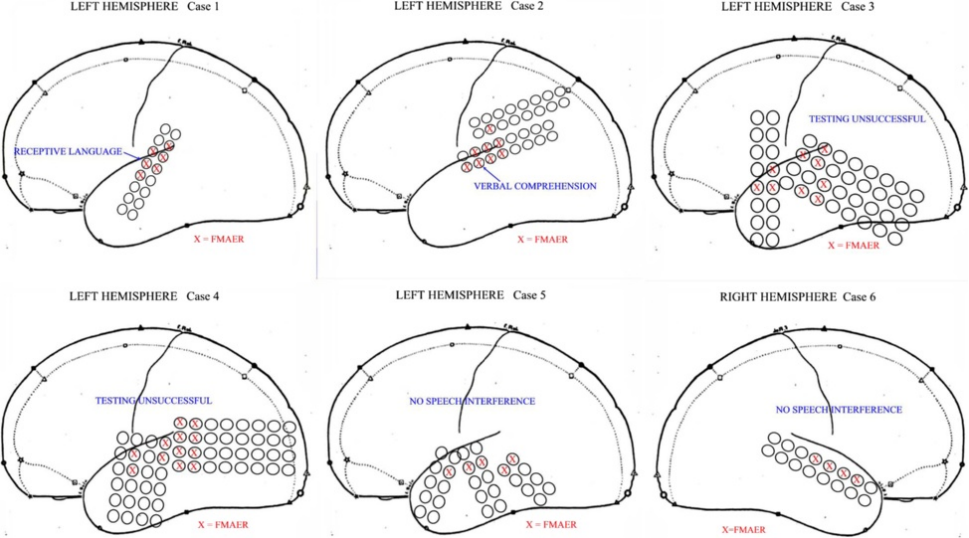
**Cortically recorded FMAER for six subjects.** Five images represent a schematic representation of the left hemisphere (Cases 1–5) and one the right hemisphere (Case 6). Atop the hemisphere images are placed representations of one or more grids, i.e. electrode contacts embedded within a thin plastic strip. Electrode centers are 1 cm apart and are used for recording EEG activity during invasive, inpatient recording and may also be utilized for FMAER recording as well as for invasive electrical stimulation to detect and localized eloquent language cortex prior to epilepsy surgery. The red crosses represent contacts where the FMAER was readily detected. For cases where cortical stimulation was undertaken, results are summarized as shown in blue font text. Note the temporal location of the FMAER responses. Cases 1 and 2 demonstrated language interference upon cortical stimulation as shown in blue text. Cases 3–6 showed no response to cortical stimulation.

Figure 7 shows schematic maps estimating grid and strip placement for six surgical patients for whom FMAER had been requested to facilitate localization of language-eloquent cortex. These studies were requested when language evaluation by direct cortical stimulation was felt to be potentially unreliable due to immaturity, behavioral difficulty, language disorder, and/or clinician-patient language barrier. This group spanned the 3–20 year old age range. Only grids involving the lateral temporal surface are shown. Grid electrode contacts showing responses following the 4 Hz FMAER stimulus are marked with a red X. Contacts that failed to respond show no X. Grid contacts in the frontal, parietal, inferior temporal or occipital regions never demonstrated FMAER responses. The boundary between contacts showing 4 Hz FMAER responses and those without response was typically quite abrupt.

Direct cortical stimulation demonstrated a small region of receptive language impairment in Case 1 within the mid left superior temporal gyrus. For Case 2 direct cortical stimulation induced receptive impairment in almost the same location. Testing by cortical stimulation was technically unsuccessful for cases 3 and 4 despite clear FMAER responses. For Case 5, no language interference was observed for cortical stimulation of any of the shown contacts. However, a delay was found in naming objects for contacts in the medial left anterior temporal base likely involving the parahippocampal gyrus and affecting memory. Case 6, where cortical recording was limited to the right hemisphere, showed as expected, no language interference anywhere within the right hemisphere. An FMAER was observed - with scalp electrodes - from the left posterior temporal region (not illustrated).

Thus, for cortical stimulation delineated language-eloquent contacts (Cases 1 and 2), co-resident corresponding FMAER responses were clearly obtained. Furthermore, for all six surgical cases, the contacts that showed FMAER responses roughly corresponded to the regions delineated in normal subjects by source analysis. However, it became clear that over the left as well as the right temporal cortex the FMAER responsive area extended beyond eloquent cortex as determined by cortical stimulation. It is unclear at this point whether this represents a response to the presence of cortical pathophysiology, such as seizure discharges, results from lack of detailed language assessment, or constitutes a normal phenomenon.

**Figure 8:**
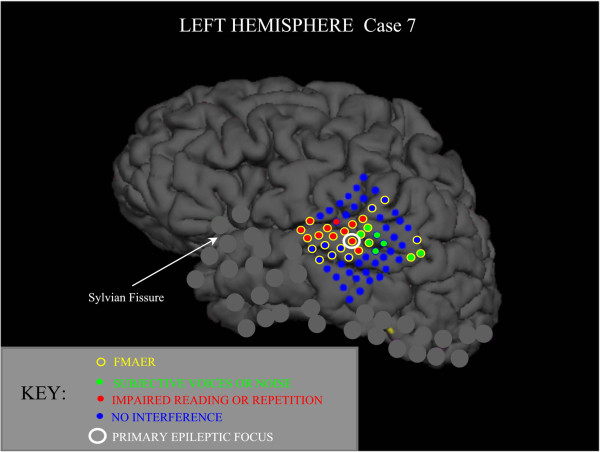
**Cortically recorded FMAER; high electrode density grid, one subject.** The true lateral surface and grid contact locations are shown as reconstructed from MRI and CT studies. The superior surface (vertex) is shown above, inferior temporal surface below, prefrontal lobe to left and occipital pole to right. An 8 × 8 (64 contact) high density grid is shown as placed over the left temporal region where contact centers are 0.5 cm apart. The FMAER was recorded from electrodes surrounded by yellow circles. Red dots signify contacts exhibiting impairment of reading and/or of speech repetition during cortical stimulation. The green dots are regions where the patient reported hearing voices or noise. The single contact showing the primary epileptic focus is surrounded by a larger white circle. Blue dots represent contacts not found to interfere with language function during cortical stimulation. Contacts of additional standard grids placed elsewhere are grayed out to avoid image clutter. None of these grayed out standard grid contacts demonstrated FMAER responses.

Figure 8 shows a single surgical patient for whom a grid with a very dense placement of electrode contacts was employed. The cortical anatomy and the grid placement as shown were reconstructed from the patient’s MRI and CT scans. The image (see legend) summarizes the results of the FMAER stimulation and the extensive cortical auditory language mapping by direct stimulation. Additional contacts on coarser grids and strips are grayed-out. None of these additional contacts demonstrated any response to FMAER or cortical stimulation. Of the eleven contacts (red) that showed impaired reading or word repetition upon direct cortical stimulation, ten showed well developed FMAER responses. Of the eight contacts (green) that resulted in the patient’s report of subjective voices or noise, five showed FMAER responses. Nine contacts showed FMAER responses yet failed to show concurrent language eloquence (yellow circles around blue contact) to cortical stimulation. The two contacts located in the upper right quadrant of the grid overlaid the angular gyrus. The detection of language eloquence there might have required more complex stimulation paradigms than were utilized. The six contacts located mainly in the upper left grid quadrant and just anterior to the indicated primary epileptic focus were found to overlay an underlying MRI detected lesion of undetermined type. When the regions of this lesion as well as the epileptic focus were surgically removed seizures ceased without obvious loss of language function.

Since preoperative scalp recorded FMAERs were not performed for any of the above patients, a direct comparison between scalp and cortical localization was not possible.

### Clinical patients with mixed receptive and expressive speech disorders

**Figure 9:**
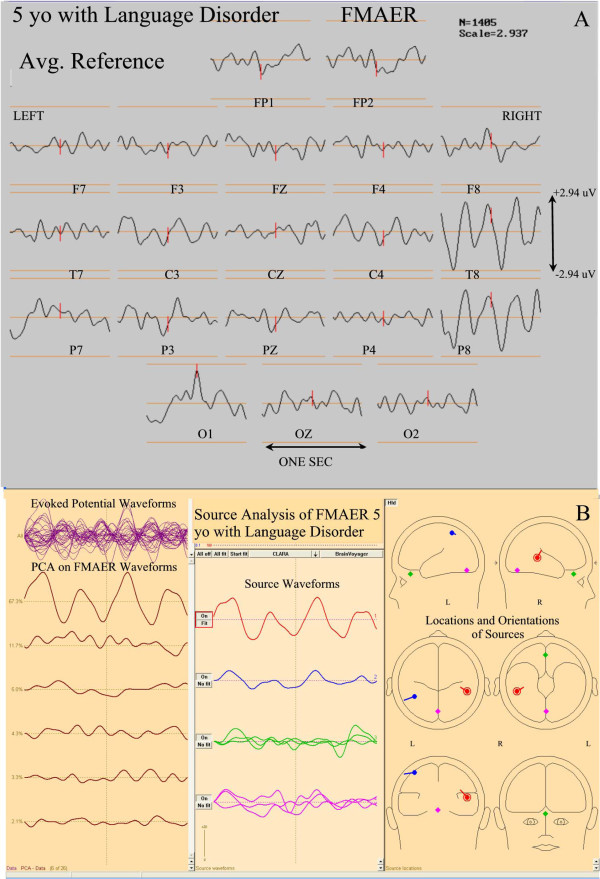
**FMAER and source analysis, 5 year old with language delay.** The top section (**A**) shows 20 channels of a scalp recorded FMAER with channel designation displayed below corresponding waveforms. Amplitude scale is to the right. The common average reference is employed. The patient demonstrated severe mixed receptive and expressive dysphasia without autistic behavioral features. Note the excellent right temporal and missing left temporal response. The bottom section (**B**), displays the FMAER source analysis results for Figure 4. Note the normally placed right temporal primary (red) source and also the aberrantly, superiorly placed and distorted left sided secondary (blue) source. There is no clear left temporal scalp response.

**Figure 10:**
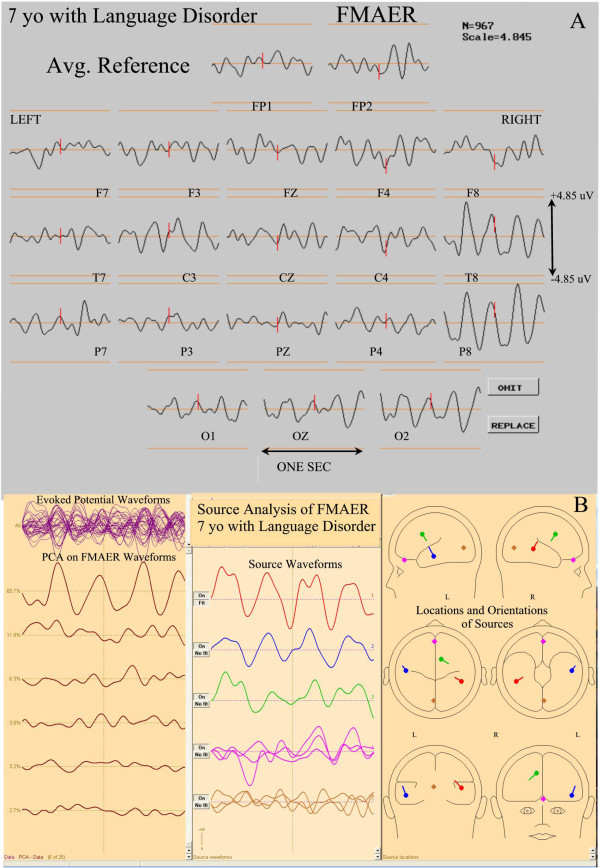
**FMAER and source analysis, 7 year old with language delay.** Display convention is as for Figure 9. The clinical presentation was also quite similar. Again note the absent left temporal scalp response, top section (**A**). Again source analysis below (**B**) shows a near normal right temporal primary response (red). The left sided secondary response (blue) shows near normal morphology but is unusual in terms of the anterior placement within the temporal lobe and the unusual source orientation. A tertiary source (green) is observed in the right medial cingulate gyrus probably related to excessive stimulus amplitude (see Hagenmuller at al. [23]).

Figures 9 and 10 show the scalp waveform appearance and source analysis results for two children with significant mixed receptive/expressive language disorder. Of note were the excellent non-dominant right temporal responses for both patients (Figures 9A and 10A), and the essentially absent left temporal responses.

For the 7 year old patient depicted in Figure 9, the right temporal source showed normal morphology as well as location and orientation (Figure 9B, red, center and right panes). While a left temporal response was clearly present, the waveform was of slightly lower amplitude (Figure 9B, blue, middle pane) than noted on the right (Figure 9B, red, middle pane). Although found within the left temporal lobe, the location and orientation of the left temporal source was quite aberrant (Figure 9B, blue source dipole, right pane). Thus, this patient’s FMAER (Figure 9A) appeared to be absent unilaterally in the scalp response display likely because of the aberrant left sided source location and aberrant source orientation.

For the 5 year old patient (Figure 10B, lower three panes) the right sided primary source was placed normally in the right posterior superior temporal gyrus and was oriented normally (Figure 10B, red source, lower middle and right panes). A weak and aberrant, very low amplitude left parietal poorly defined 4 Hz response was present. A left temporal 4 Hz response was not seen. (10B, blue source, middle and right panes).

**Figure 11:**
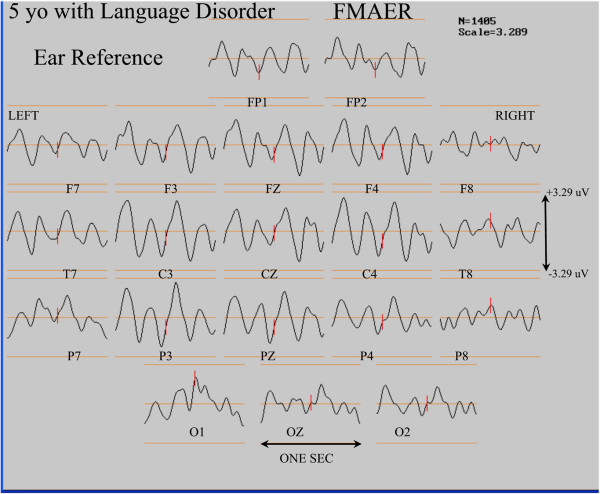
**Effect of EAR reference on FMAER with absent left sided response.** The display convention is as for Figure 9A. Note that redisplay of the Figure 9A data utilizing ear reference causes the restricted right sided response (Figure 9A) to now appear widely distributed even including the left temporal region (Hagenmuller at al. [23]).

In Figure 11 the 5 year old patient’s FMAER data displayed in Figure 9A are also shown when the ear reference method was used. Note the marked difference between the common average and ear reference conditions in the FMAER’s spatial distribution. The common average reference (Figure 9A) demonstrated a highly localized response in the right temporal regions (T8, P8) whereas the use of the ear reference resulted in a broad, bilateral pattern which made visual detection of the missing left temporal response highly problematic.

### A patient with Landau-Kleffner syndrome before and after successful treatment

**Figure 12:**
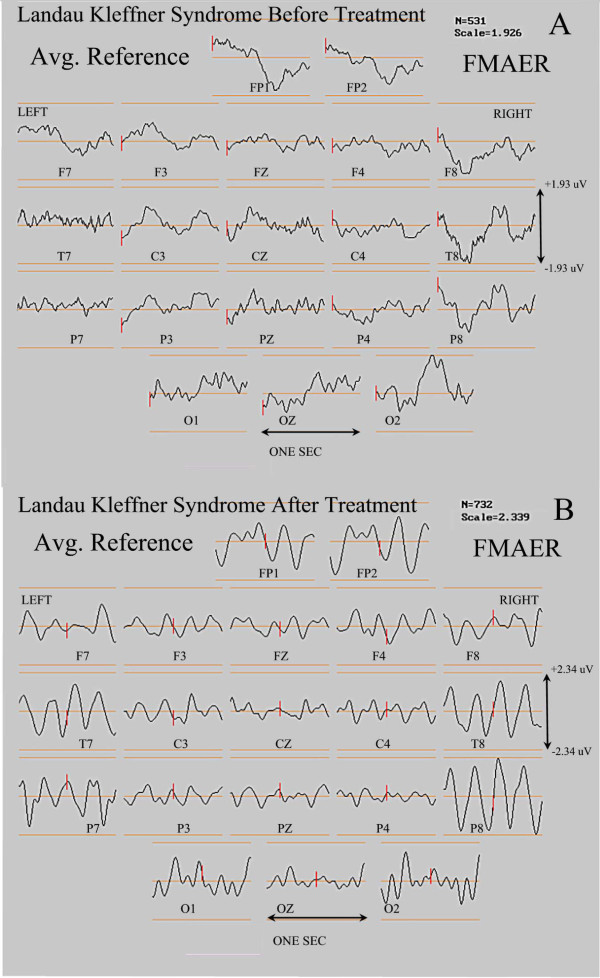
**FMAER, Landau-Kleffner syndrome before and after treatment.** Display convention is as for the upper portions of Figures 9 and 10. The top (**A**) shows absence of an FMAER response over either hemisphere before treatment. The bottom pane (**B**) shows presence of normal bilateral temporal responses 13 months later after pharmacological treatment. Language had dramatically improved by the time of the second study (**B**).

Figure 12 shows the FMAER scalp topographies for an initial study (top pane, 12 A) at the time of LKS diagnosis. Language, both receptive and expressive, had significantly declined. Although there were no clinical seizures, the waking EEG demonstrated bilateral anterior and independent left and right temporal seizure discharges (spike waves). Also there was a marked accentuation of discharges in sleep consistent with electrical *status epilepticus* of sleep (ESES). The FMAER was bilaterally absent (12 A). Approximately 13 months later, after treatment with nocturnal benzodiazepine, daytime lamotrigine, and daily prednisolone significant improvement in all aspects of language was observed. The EEG failed to demonstrate discharges in waking. In sleep there were only a few scattered discharges and ESES was absent. FMAER responses were clearly obtained in both temporal regions (bottom pane, 12 B).

This case illustrates the FMAER’s utility for the identification of physiologic correlates of language improvement. The functional improvement in language was clearly accompanied by marked FMAER improvement.

### A patient with Landau-Kleffner syndrome and evidence for rapidly declining function

**Figure 13:**
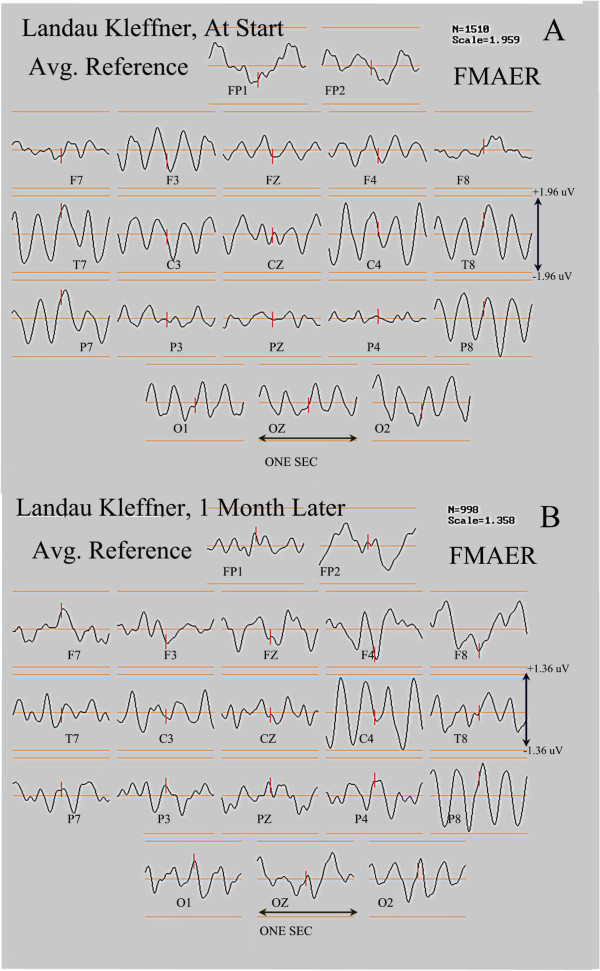
**FMAER, Landau-Kleffner syndrome, deterioration over one month.** The FMAER is shown from a second subject with the Landau-Kleffner syndrome. Display convention is as for Figure 12. The top display shows a normal FMAER response at the time the patient was first studied, presenting with behavioral issues and poor verbal memory without dysphasia. One month later, the left temporal FMAER response had clearly deteriorated and the patient demonstrated receptive language difficulty. See text and Figure 14 for more detail.

Figure 13 shows the FMAER of an adolescent patient who presented with behavioral irritability and declining school performance. The patient showed no clear receptive or expressive dysphasia, however the patient had great difficulty remembering auditory information. The EEG demonstrated left central-parietal spikes with occasional spontaneous electrographic seizures (over ten seconds in length) with focal left central-parietal (CP5) spikes. The FMAER demonstrated a normal bilateral response (Figure 13A). One month later the patient demonstrated further clinical deterioration with slowed speech production and receptive greater than expressive dysphasia. At that time the repeat FMAER demonstrated a clear left temporal deficit (Figure 13B).

**Figure 14:**
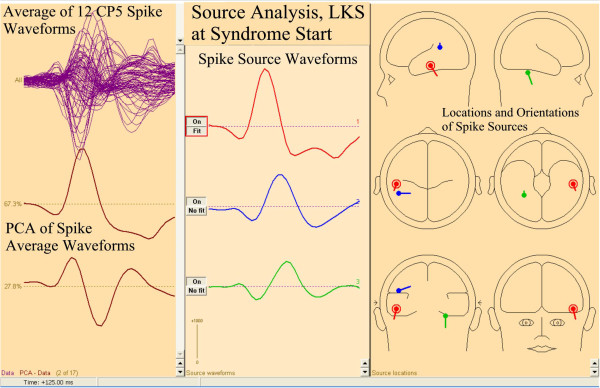
**FMAER, Landau-Kleffner syndrome, CP5 spike source analysis.** Source analysis for the patient described in Figure 13 is shown for the average of 12 CP5 (left parietal-temporal) spikes during a short electrographic seizure and at the time of the initial study. The patient’s FMAER is shown in Figure 13A. Display convention for Figure 14 is as for Figure 4 although data represent the CP5 spike average. Waveforms for the three sources are shown in the middle pane with the corresponding source locations and orientations shown in the right hand pane. The primary source is shown in red, secondary source in blue, and tertiary source green. Note (middle pane) that the primary source leads the other sources in time and is located in the basilar temporal region. Note that the secondary lower amplitude and later occurring source is located in the left angular gyrus. The time scale for all waveforms is 125 msec.

Source analysis of an average of 12 CP5 spikes from the time of the first study demonstrated the primary spike source (Figure 14, red source, right pane) to be located in the left temporal base and possibly related to the memory difficulty. A secondary spike source was located in the left Wernicke’s region (Figure 14, blue source, right pane), which may relate to the subsequent language deterioration. An additional tertiary source was seen in the right medial temporal base (Figure 14, green source, right pane).

This case illustrates the FMAER’s ability to identify physiological deterioration accompanying progression of LKS. The functional deterioration was clearly associated with FMAER deterioration.

### A summary of findings for 18 subjects with LKS and/or ESES

Table 1 summarizes the findings for 18 right-handed patients carrying the clinical diagnosis of LKS, ESES, or most commonly both diagnoses. All patients had received both FMAER studies and overnight EEG monitoring by in-hospital or ambulatory recordings. Ages ranged from 5 to 15 years. All patients presented with declines in cognition, behavior, memory, and language. The type and degree of language abnormality was typically referred to as mixed receptive/expressive dysphasia. FMAER studies were performed prior to overnight monitoring and subsequent institution of pharmacological therapy. EEG epochs containing prominent artifact or discharges were eliminated from EEG segments used to form the FMAER.

**Table 1:** Patients with LKS and/or ESES - earliest available study

**Case No.**	FMAER*	**ESES**	**Generalized discharges**	**Focal discharges**	**Source in STG**	
					**Left**	**Right**
**1**	abnml B	50%	Yes	Yes: T7, T8, P7, P8	yes	yes
**2**	abnml B	90%	Yes	Yes: T7, T8	yes	yes
**3**	abnml B	0%	Yes	Yes: P7, P8	yes	yes
**4**	abnml B	0%	No	Yes:● T7, P7, T8, P8	yes	yes
**5**	abnml B	40%	No	Yes: CP5, C3	yes	n/d
**6**	abnml B	0%	Yes	Yes:● CP5, C3	yes	n/d
**7**	abnml B	60%	Yes	Yes: P7, O1, P8	yes	yes
**8**	abnml B	43%	Yes	Yes: CP5, C3, P3, P4	yes	yes
**9**	abnml B	80%	Yes	Yes: P7, P8	yes	yes
**10**	abnml L	98%	Yes	Yes: P7, O1, P8, O2	yes	no
**11**	abnml L	90%	Yes	Yes: T7, T8	yes	no
**12**	abnml B	89%	Yes	Yes: CP5, P7 P8	yes	yes
**13**	abnml B	35%	No	Yes: CP5, C3, P3	yes	n/d
**14**	normal	90%	Yes	Yes: P4, P8	no	no
15**	normal	75%	Yes	Yes:● CP5, FC5, T7, F7	(no)	n/d
**16**	normal	0%	Yes	Yes: FC5, C3	((no))	n/d
**17**	normal	59%	Yes	Yes: O2, O1, FC2, C4	no	no
**18**	normal	58%	Yes	Yes: CZ, PZ, C4, P4	no	no

Legends:

* = “abnml” indicates abnormal (absent or severely distorted) FMAER; B = bilaterally; L = left side only (right side only cases not found).

** = Case 15 FMAER became abnormal 1 month after initial study (see Figures 13 and 14).

● = FMAER stimulation reported to mildly activate left temporal discharges.

**ESES** = Percent of seconds of Stage 2 sleep containing discharges. Not all reach typical ‘80% definition’ for ESES but value shown reported as ‘clinically significant’.

**Source in STG** = Primary source for source analysis of focal discharges found in superior temporal gyrus and/or Wernicke’s area - ‘yes’ or ‘no’.

‘n/d’ = Not done, insufficient discharges present for source analysis.

(no) = primary source found in mid-inferior left temporal region but a prominent *secondary* source noted in left superior temporal gyrus.

((no)) = Source analysis of FC5 spikes shows primary source in frontal lobe near Broca’s area, distant from the posterior temporal region.

The FMAER was considered abnormal when absent or distorted, either bilaterally or unilaterally. Of the 18 patients 13 showed abnormal FMAERs; 11 of the 13 showed bilateral and two showed exclusively left sided abnormalities. Unilateral right sided abnormalities were not observed.

Source analyses of the left and right temporal spike averages were performed in order to determine whether the primary source component involved the region of the corresponding side’s superior temporal gyrus and/or Wernicke’s gyrus. Table 1 additionally notes the presence or absence of generalized discharges, the location of focal discharges and the spike wave index for overnight recording.

Cases 4, 5, and 13 might be considered primary LKS without ESES since no generalized discharges were observed even in sleep. Cases 5 and 13 demonstrated focal EEG activation during sleep in contrast to generalized spike wave activation during sleep, observed for other cases. Cases 3, 4, 6, and 16 failed to demonstrate any sleep potentiation of discharges. In cases 4, 6, and 15 EEG during FMAER stimulation showed increased frequency of left temporal discharges. However, in these three cases the FMAER failed to initiate clinical or electrographic seizures.

Note that all of the cases with abnormal FMAERs (Cases 1–13) source analysis demonstrated left hemisphere EEG discharges with demonstrable primary sources in or close to the posterior superior left temporal region. Eight of the 13 cases also manifested epileptiform involvement of the homologous right side (Cases 1–4, 7–9, 12). Three (Cases 5, 6, 13) failed to show right sided discharges which obviated right sided source analysis. The two cases showing unilateral left sided FMAER abnormality (Cases 10, 11) did not show right posterior superior temporal sources.

Similarly, note that none of the studies with normal FMAERs (Cases 14–18) demonstrated primary sources in the left or right posterior temporal regions - two (Cases 15, 16) showed no right sided discharges for analysis. Case 15 (whose data are shown in Figures 13 and 14) showed a prominent *secondary* source in the vulnerable left posterior temporal region during the first recorded study at a time when the FMAER remained normal. One month later, however, when the clinical symptoms had progressed, the left FMAER had become abnormal (Figure 13B). A source analysis at the time of the second study was not possible since discharges were not recorded during the second study.

These cases demonstrate that the bilateral absence of the FMAER is primarily associated with the presence of focal discharges originating in or near the bilateral superior temporal gyri. Absence of just the left sided FMAER was associated with just left sided spike source involvement. Those with normal bilateral FMAERs did not show primary spike source involvement in either of the superior temporal gyri.

### Two patients with autism spectrum disorder

**Figure 15:**
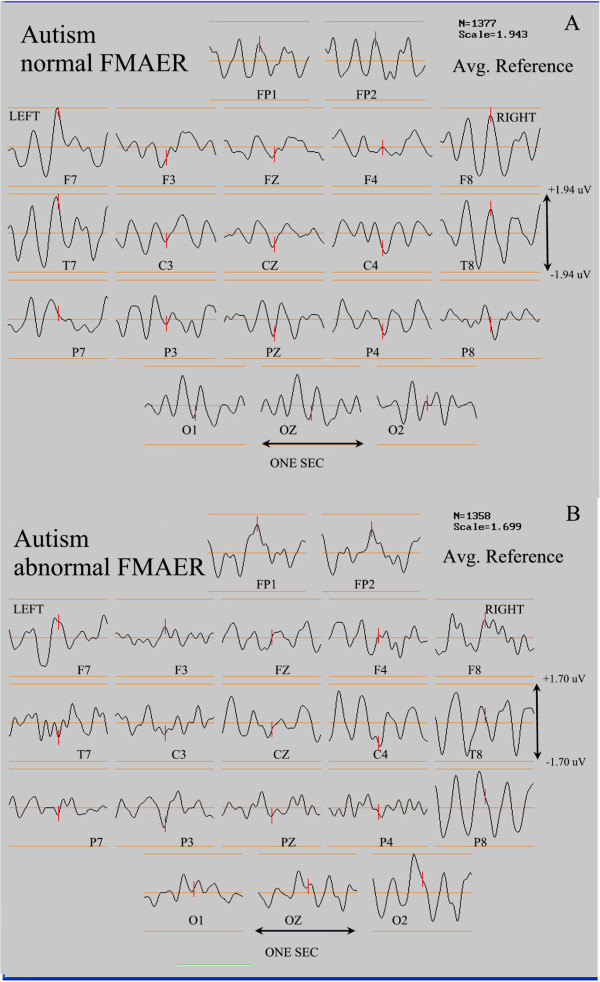
**Normal and abnormal FMAERs in autism.** The display convention is as for Figure 12. In the current figure, the top pane (**A**) illustrates a normal FMAER in a patient with autism. The bottom pane (**B**) shows an abnormal FMAER that is absent in the left temporal region of another patient with the same diagnosis of autism (see text for discussion).

Figure 15 shows the scalp FMAER study results from two patients diagnosed with autism spectrum disorder. Both patients had marked language impairment. One patient (Figure 15A) demonstrated near normal bilateral FMAER scalp responses. Despite the patient’s severe mixed language impairment, he frequently and clearly mimicked parts of speech yet in a nonsensical, jargon-like manner. The other patient (Figure 15B), who also demonstrated severe mixed language impairment, failed to produce word-like sounds of any kind; his vocal output exclusively involved grunts and screeches. This patient (Figure 15B) demonstrated an excellent right sided response; his left sided response was absent. These two cases illustrate the FMAER’s potential utility in ASD without functional language. Partial speech component production was accompanied by an intact FMAER, while complete absence of all speech production was accompanied by an absent FMAER.

## Conclusions

This report demonstrates the potential utility of the FMAER for study of children with language disorders. Six advances result from the investigation:

*First*, the findings constitute the first demonstration by use of discrete source analysis that the FMAER arises symmetrically from the bilateral posterior superior temporal lobes for neurotypical controls and patients with normal language function. Involved areas include primarily the *planum temporale*, posterior superior temporal gyrus, and to a more variable extent the middle temporal gyrus. These locations were consistently identified across all ten neurotypical subjects evaluated as well as the five clinical subjects with normal language function (Figure 6). Moreover, when FMAER data were obtained directly from the cortex of patients undergoing Phase 2 pre-surgical epilepsy evaluations, the same general cortical regions appeared active (Figures 7 and 8).

*Second*, for epilepsy surgical cases the cortically recorded FMAER delineates putative language eloquent cortex should such delineation prove difficult with traditional techniques of cortical stimulation for various reasons as discussed above (see Figures 7 and 8). Cortical FMAER localization is generally consistent with the regions predicted by FMAER source analysis in subjects with normal language function.

*Third*, this investigation illustrates that use of reference electrodes near active temporal regions such as the ear lobes or mastoid processes distort scalp topographic morphology when scalp FMAER recordings are employed. For example, Figures 9 and 11 illustrate the absence of a left temporal response when the common average reference was utilized (Figure 9A). Moreover, the important identification of a missing response was obscured when ear referencing was employed (Figure 11). Thus, typical laboratory use of frontal to mastoid recordings should be avoided. As the investigations demonstrate, use of frontal to ear or mastoid recordings produces a large signal because the two recording electrodes (frontal and mastoid) cross the temporal dipole. However, because both left and right superior temporal gyri contribute to the wide frontal response, a unilaterally missing temporal source is likely to remain undetected. In order to correctly utilize reference free recording techniques, multiple scalp electrodes should be utilized of which at least some should be placed in the sub-temporal regions [28,31].

*Fourth*, two patients with developmental dysphasia, both of whom showed absent left sided FMAERs, were selected as examples for this report in order to demonstrate and resolve apparently contradictory findings. One patient (Figure 7) showed surface recordings that suggested an absent left temporal response when, in fact, a left sided source was present upon source analysis yet was aberrant in location and orientation. The second patient also showed an absent left temporal FMAER by surface recording which, however, was validated by source analysis (Figure 5). In certain instances, source analysis appears to be helpful in facilitating the full understanding of FMAER findings. The clinical value of the FMAER remains to be investigated further in comparison to conventional clinical and neurophysiological evaluations of developmental dysphasia.

*Fifth*, two illustrative cases were provided in order to show that LKS patients may manifest normal, unilaterally abnormal, or bilaterally abnormal FMAERs depending upon the state of severity of their illness. The FMAER may worsen when the illness progresses (Figure 13) and improve when pharmacotherapy is successful (Figure 12). Whether such findings are universal for LKS and/or whether the FMAER shows added value for clinical use with LKS patients remains to be investigated.

*Sixth*, a small case study of patients with LKS/ESES shows that the FMAER is abnormal only when temporal discharges are present and the primary epileptic source, as identified by source analysis of these discharges, is observed to reside in or near the posterior superior temporal gyrus. Eleven of the 13 abnormal studies demonstrated bilateral abnormality consistent with the Lewine et al. report [32] of magnetoencephalogram (MEG) spikes in both superior temporal gyri of LKS patients, and the Takeoka et al. report [33] of bilateral volume reduction of these regions in LKS.

The curious lack of isolated right sided FMAER abnormalities may reflect the patient selection criteria, which were based, almost entirely, upon manifest language dysfunction and therefore typically suggestive of left hemisphere dysfunction. Recently, transcranial magnetic stimulation (TMS) studies by Harpaz et al. have demonstrated [34] that the right Wernicke’s area seems to be specifically involved in *higher* language functions. These investigators show data suggesting that this right sided region processes “subordinate meanings” of ambiguous words. For example the word “bank” has a different meaning in association with the words “river” or “teller”. Such subtle language dysfunction may not have brought children in for study or may have been subsumed under the “global decline” diagnosis often reported with early LKS/ESES.

Another question involves how to explain language loss in cases when the FMAER is present (Cases 14–18) and the bilateral Wernicke’s regions are therefore assumed to be functional. An answer may lie in the findings outlined by Newman et al. [35] which indicate that the range of cortical areas implicated in speech processing goes well beyond the classical cortical regions typically involved in language. These authors demonstrated that wide frontal areas including Broca’s area and broader inferior temporal regions may be involved in functions typically attributed to Wernicke’s area. Thus generalized as well as focal discharges not involving Wernicke’s areas may be implicated in language deterioration. These questions deserve further study.

*Seventh*, of two patients with ASD one had a normal FMAER (Figure 15A) and the other was missing a left temporal response (Figure 15B). Although apparently equally language impaired, the patient with the normal FMAER showed clinical evidence of some receptive language competence (Figure 15A), incorporating phonemes into his babbling whereas the other showed no clinical evidence of even such rudimentary receptive language competence (Figure 15B). Of note, Saygin [36] demonstrated in adult aphasic patients that lesions involving Wernicke’s area and the superior temporal gyrus predicted deficits in processing of both non-verbal as well as verbal sounds. The value of the FMAER in autism remains to be evaluated further.

On the basis of outlining the technical refinements necessary for successful FMAER administration and analysis and the demonstration of consistent FMAER source localization, the information presented suggests that the FMAER may constitute a sensitive test for the clinical study of childhood language disorders and, in particular, a probe of function within both left and right posterior superior temporal gyri. The FMAER may well be very helpful in the assessment of the capacity of bilateral posterior superior temporal auditory cortex to process frequency modulated signals, a very basic component of spoken human language. Whether the FMAER will be shown to provide added value to currently available laboratory tests and clinical expertise remains to be demonstrated. Detailed studies to detect and assess the FMAER’s potential value in both outpatient clinics and inpatient services are indicated.

In the same sense that the MRI is successfully employed by many clinicians with no detailed knowledge of underlying MRI physics, it is certainly possible for clinicians to successfully employ the FMAER without a full understanding of the role of FM in language. It is only necessary to understand that the FMAER constitutes a probe of the bilateral posterior-temporal language regions and does not assess language function outside of these areas. The FMAER is not yet ‘diagnostic’ for any known disease. Clinicians wishing to employ the technique must know that for scalp recordings a full head of electrodes, including subtemporal electrodes, is best and that evaluation should be done on the common average or Laplacian references, avoiding ear/mastoid references. The clinician should know that the FMAER may be bilaterally or unilaterally normal, absent, or more rarely distorted. The need to supplement studies with source analysis is likely to be infrequent but this technique may sometimes prove helpful.

Invasively, cortical FMAER may assist in the elucidation of language eloquent cortex although the FMAER-negative grid contacts cannot be assumed to have no language function as the FM component of language – although important and widespread– is by no means a constituent of every aspect of hierarchical language processing.

Subsequent investigations will be required to delineate the spectrum of clinical FMAER indications beyond, LKS and invasive surgery, in children as described herein.

## Abbreviations

AER: Auditory evoked response;AM: Amplitude modulation;ASD: Autism spectrum disorder;BCH: Boston Children’s Hospital;DNL: Developmental Neurophysiology Laboratory at BCH;EEG: Electroencephalograph, Electroencephalography;ECoG: Electrocorticogram, Electrocorticography;ESES: Electrical *status epilepticus* of sleep;fMRI: functional MRI;ER: Evoked response;FM: Frequency modulation;FMAER: Frequency modulated auditory evoked response;IRB: Institutional Review Board of BCH;LKS: Landau-Kleffner syndrome;MEG: Magnetoencephalogram, Magnetoencephalography;MRI: Magnetic resonance imaging;PCA: Principal Components Analysis;SPL: Sound Pressure Level;TMS: Transcranial magnetic stimulation;Vrms: Root mean square voltage

## Competing interests

The authors declare that they have no competing interests.

## Authors’ contributions

All authors contributed extensively to the study’s concept and design including the range of topics to be presented. FHD and AS in collaboration with YZE, AR, and HA selected specific research subjects and clinical patients to be illustrated. JRM performed the neurosurgical procedures including selective placement of grids and strips for the epilepsy patients. FHD performed all source analyses and reported all clinical FMAER studies. Collectively FHD, YZE, AR, and JRM were involved in the clinical evaluation of all epilepsy patients. FHD, YZE, and AR participated in the direct stimulation of grids and strips. FHD supervised all clinical and research data collection and analysis, had full access to all study data, and takes full responsibility for all aspects of the study including data integrity and accuracy. All authors collaborated in writing and editing the paper and approved the final manuscript.

## Authors’ information

FHD is a physician, child neurologist, electroencephalographer, neurophysiologist and electrical engineer. Current research interests are in neuro-developmental disorders and epilepsy, including the development and utilization of specialized techniques to support related investigations. YZE is a physician, child neurologist, epileptologist, electroencephalographer, and clinical and research neurophysiologist. AR is a physician, child neurologist, epileptologist, electroencephalographer, and clinical and research neurophysiologist. Research interests include transcranial magnetic stimulation and its application to epilepsy and neurodevelopmental disorders. JRM is a physician, neurosurgeon, and neurophysiologist with special interests in epilepsy surgery and source localization including localization of eloquent sensory cortex. AS is a cognitive neuroscientist who has specialized interests in EEG and its identification of neuro-developmental disorders and particularly developmental language disorders. HA is a research and clinical psychologist with interest in infant and child neurodevelopment, including the generating of predictors of later outcome from neurophysiologic data.

## Pre-publication history

The pre-publication history for this paper can be accessed here:

http://www.biomedcentral.com/1471-2377/13/12/prepub

## References

[B1] Green GGR Kay RH Rees A Responses evoked by frequency-modulated sounds recorded from the human scalp J Physiol 1979 296 21 22P 529087

[B2] Green GGR Rees A Stefanatos GA A method for recording evoked responses to frequency modulated sounds in man J Physiol 1980 307 10p

[B3] Stefanatos GA Green GGR Ratcliff GG Neurophysiological evidence of auditory channel anomalies in developmental dysphasia Arch Neurol 1989 46 August 871 875 2757527 10.1001/archneur.1989.00520440053021

[B4] Stefanatos GA Frequency modulation (FM) analysis in children with Landau-Kleffner syndrome Ann N Y Acad Sci 1993 682 412 414 10.1111/j.1749-6632.1993.tb23009.x 8323151

[B5] Tomblin JB Abbas PJ Records NL Brenneman LM Auditory evoked responses to frequency-modulated tones in children with specific language impairment J Speech Lang Hear Res 1995 38 2 387 392 10.1044/jshr.3802.387 7596104

[B6] Stefanatos GA Foley C Grover W Doherty B Steady-state auditory evoked responses to pulsed frequency modulations in children Electroencephalogr Clin Neurophysiol 1997 104 31 42 10.1016/S0168-5597(96)96042-6 9076251

[B7] Talcott JB Witton C McClean M Hansen PC Rees A Green GG Stein JF Can sensitivity to auditory frequency modulation predict children’s phonological and reading skills Neuro Report 1999 10 10 2045 2050 10.1097/00001756-199907130-00010 10424672

[B8] Tallal P Stark R Curtiss B The relation between speech perception impairment and speech production impairment in children with developmental dysphasia Brain Lang 1976 3 2 305 317 10.1016/0093-934X(76)90025-0 938935

[B9] Tallal P Language and reading: some perceptual prerequisites Bulletin Orton Society 1980 30 170 178 10.1007/BF02653716

[B10] Tallal P Start RE Kallman C Mellits D Developmental dysphasia: Relation between acoustic processing deficits and verbal processing Neuropsychologia 1980 18 273 284 10.1016/0028-3932(80)90123-2 6158017

[B11] Tallal P Galaburda A Llinas R von Euler C Temporal information processing in the nervous system: Special reference to dyslexia and dysphasia (Volume 682 of the Annals of the New York Academy of Sciences) 1993 New York, NY: New York Academy of Sciences 8100695

[B12] Tallal P Neurobiological basis of speech: a case for preeminence of temporal processing Ann N Y Acad Sci 1993 682 27 47 10.1111/j.1749-6632.1993.tb22957.x 7686725

[B13] Wright BA Lombardino LJ King WM Puranik CS Leonard CM Merzenich MM Deficits in auditory temporal spectral resolution in langiage-impairted children Nature 1997 387 6629 176 178 10.1038/387176a0 9144287

[B14] Duffy FH McAnulty GB Waber DP Auditory evoked responses to single tones and closely spaced tone pairs in children grouped by reading and matrices abilities Clin EEG 1999 30 84 93 10.1177/155005949903000303 10578470

[B15] Nagarajan S Mahncke H Salz T Tallal P Roberts T Merzenich MM Cortical auditory signal processing in poor readers Proc Natl Acad Sci 1999 96 11 6483 6488 10.1073/pnas.96.11.6483 10339614 PMC26908

[B16] Stefanatos GA Speech perceived through a damaged temporal window: Lessons from word deafness and aphasia Semin Speech Lang 2008 29 3 239 252 10.1055/s-0028-1082887 18720320

[B17] Kay RH Matthews DR On the existence in human auditory pathways of channels selectively tuned to the modulation present in frequency modulated tones J Physiol 1972 225 657 677 5076392 10.1113/jphysiol.1972.sp009962 PMC1331136

[B18] Regan D Tansley BW Selective adaptation to frequency modulated tones: evidence for an information-processing channel sensitivity to frequency changes Journal of the Acoustic Society of America 1979 65 1249 1257 10.1121/1.382792 458046

[B19] Jayakar P Alvarez LA Duchowny MS Resnick TJ A safe and effective paradigm to functionally map the cortex in childhood J Clin Neurophysiol 1992 9 288 293 10.1097/00004691-199204010-00009 1592899

[B20] Zangaladze A Sharan A Evans J Wyeth DH Wyeth EG Tracy JL Chervoneva J Sperling MR The effectiveness of low-frequency stimulation for mapping cortical function Epilepsia 2008 49 3 481 487 10.1111/j.1528-1167.2007.01307.x 17868054

[B21] de Ribaupierre S Fohlen M Bulteau C Dorfmueller G Delanda O Chiron C Hert-Pannier L Presurgical language mapping in children with epilepsy: clinical usefulness of functional magnetis resonance imaging for planning of cortical stimulation Epilepsia 2012 53 1 67 78 10.1111/j.1528-1167.2011.03329.x 22126260

[B22] Van Drongelen W Signal Processing for Neuroscientists: an Introduction to the Analysis of Physiological Signals vol. 5 2011 Oxford: Elsevier

[B23] Hagenmuller F Hitz K Darvas F Kawohl W Determination of the loudness dependence of auditory evoked potentials: single-electrode estimation versus dipole source analysis Hum Psychopharmacol Clin Exp 2011 26 2 147 154 10.1002/hup.1186 21455973

[B24] Bartels PH **Numerical evaluation of cytologic data VIII.** Computation of the principal components Anal Quant Cytol 1981 3 2 83 90 7020519

[B25] Jackson DA Stopping values in principal components analysis: a comparison of heuristical and statistical approaches Ecology 1993 74 8 2204 2214 10.2307/1939574

[B26] Guttman L Some necessary conditions for common factor analysis Psychometrika 1954 19 149 161 10.1007/BF02289162

[B27] American Clinical Neurophysiology Society Guideline 5: Guidelines for standard electrode position nomenclature J Clin Neurophysiol 2006 23 107 110 10.1097/00004691-200604000-00006 16612226

[B28] Kayser J Tenke C In search of the Rosetta Stone for scalp EEG: Converging on reference-free techniques Clin Neurophysiol 2010 121 12 1973 1975 10.1016/j.clinph.2010.04.030 20566375 PMC2953588

[B29] Duffy FH Burchfiel JL Lombroso CT Brain electrical activity mapping (BEAM): A method for extending the clinical utility of EEG and evoked potential data Ann Neurol 1979 5 309 321 10.1002/ana.410050402 443765

[B30] Cooley WW Lohnes PR Multivariate Data Analysis 1971 New York: J. Wiley and Sons

[B31] Nunez PL Electric Fields of the Brain 1981 New York: Oxford University Press

[B32] Lewine JD Andrews R Chez M Patil A Devinsky O Smith M Kanner A Davis JT Funke M Jones G Magnetoencephalographic patterns of epileptiform activity in children with regressive autism spectrum disorders Pediatrics 1999 104 405 418 10.1542/peds.104.3.405 10469763

[B33] Takeoka M Riviello JJ Duffy FH Kim F Kennedy DN Makris N Caviness VS Holmes GL Bilateral volume reduction of the superior temporal areas in Landau-Kleffner syndrome Neurology 2004 63 1289 1292 10.1212/01.WNL.0000140703.63270.9D 15477555

[B34] Harpaz Y Levkowitz Y Lavidor M Lexical ambiguity resolution in Wernicke’s area and its right homologue Cortex 2009 45 9 1097 1103 10.1016/j.cortex.2009.01.002 19251255

[B35] Newman AJ Supulla T Hauser P Newport EL Bavelier D Dissociating neural subsystems for grammar by contrasting word order and inflection Proceedings National Academy of Sciences USA 2010 107 16 7539 7544 10.1073/pnas.1003174107 PMC2867749 20368422

[B36] Saygin AP Dick F Wilson SM Dronkers NF Bates E Neural resources for processing language and environmental sounds: Evidence from aphasia Brain 2003 126 928 945 10.1093/brain/awg082 12615649

